# Selected bacteria in sheep stool depending on breed and physiology state

**DOI:** 10.1038/s41598-023-38785-4

**Published:** 2023-07-20

**Authors:** Paulina Cholewińska, Natalia Szeligowska, Konrad Wojnarowski, Paulina Nazar, Monika Greguła-Kania, Andrzej Junkuszew, Witold Rant, Aurelia Radzik-Rant, Anna Marcinkowska, Robert Bodkowski

**Affiliations:** 1 Chair for Fish Diseases and Fisheries Biology, Ludwig-Maximilians-University of Munich, 80539 Munich, Germany; 2 Department of Chemical Sciences, University of Napoli Federico II, Via Cintia, 80126 Naples, Italy; 3 Department of Animal Breeding and Agriculture Advisory, Faculty of Animal Sciences and Bioeconomy, University of Life Sciences in Lublin, 20-950 Lublin, Poland; 4 Institute of Animal Breeding, Warsaw University of Life Sciences-SGGW, 02-786 Warsaw, Poland; 5 Institute of Animal Breeding, Wroclaw University of Environmental and Life Sciences, 51-630 Wroclaw, Poland

**Keywords:** Microbiology, Animal physiology

## Abstract

One of the important factors influencing the microbial community of ruminants, besides environment or diet, are breed and physiology. Therefore, the purpose of this study was to assess these changes in the levels of basic microbial phyla and families. For this study, qPCR analysis was performed to determine the level of bacteria (Firmicutes, Bacteroidetes, Actinobacteria, Proteobacteria clusters and *Clostridiaceae*, *Lactobacillaceae* families) in the feces of ewes of three native Polish sheep breeds (Polish Lowland Sheep (PON), Świniarka Sheep (SW), and synthetic line BCP) at different physiological periods (conception, early pregnancy, lambing, end of lactation). The animals were kept in the same environment and were at the same age (2-years). The results showed a significant effect of both breed (*p* = 0.038) and physiological period (*p* < 0.05, *p* < 0.01) on the levels of bacteria analyzed. The breed showed differences across physiological periods. The influence of the race factor was noted primarily between the BCP synthetic line and the other two breeds (differences in terms of all analyzed clusters and families except Actinobacteria phyla). In the case of SW and PON, however, the observed differences were only at the level of Proteobacteria cluster and *Clostridiaceae* family. On the other hand, the early pregnant and lambing periods were the most microbiologically diverse in terms of the analyzed clusters and families of bacteria.

## Introduction

The microbiome of the gastrointestinal tract of ruminants consists of bacteria, archaea, fungi and protozoa, forming a peculiar ecosystem. The most abundant are anaerobic or relatively aerobic bacteria, belonging to the Firmicutes, Bacteroidetes, Proteobacteria and Actinobacteria cluster. In the forestomach microbial community is the most populated, followed by the large intestine. In addition, the large intestine has a microbial population similar to the level in the rumen^1^. These bacteria are involved in the decomposition of plant particles and their conversion into energy for the animal. The products formed in these processes are volatile fatty acids (VFAs), which are the main source of energy (accounting for about 70% of requirements) and have a direct effect on the physiological parameters of the animal, such as, for example, development, health, production rates^2–5^. Analyses of microbial variability allow increasing both productivity and breeding intensity while maintaining a good animal and environment, which is necessary due to the increased demand for animal products. In addition, it will enable better manipulation of the animal microbiome to prevent the use of anti-pathogenic substances (such as antibiotics). Overuse of these in recent years has adversely affected both animals and humans, which is related to the concept of One Health^5–7^.

Over the course of life in ruminants, changes in microbial structure can occur depending on the age as well as the physiological state of the animal. One of the most stressful conditions in adult females can be pregnancy. In the case of ruminants, it has to take place every year, and is tied to both meat and milk production, depending on the type of production. The state of pregnancy in the case of sheep lasts about 5 months and is a time of dynamic physiological changes, in turn, the birth itself is a factor that causes stress in the female^8^. Given that the microbial community is a specific type of immune system that mainly prevents the growth of pathogenic microorganisms (often belonging to the *Clostridiaceae* family), stress can negatively affect its composition causing dysbiosis. The occurrence of microbial dysbiosis of the gastrointestinal tract, resulting in an increased risk of bacterial infections of both the gastrointestinal tract and others (such as bacterial vaginitis)^8,9^. An additional factor that can affect the level of dysbiosis and the overall composition of the microbial community can be the breed of animal. In the case of sheep, there are breeds selected for their meat, milk or wool production. In addition, a selection factor has been to increase resistance to environmental conditions, which has also resulted in the emergence of many local breeds or lines^1,10–12^.

Studies conducted in recent years by Douglas et al.^13^ or Xin et al.^14^ support the theory that there are differences in the microbial composition of the gastrointestinal tract in different breeds of ruminants—both cattle and sheep. It has been suggested that different diets were used to achieve the results expected by breeders and were maintained for many years in different environments or housing systems. Selection under factors such as maintenance system or diet may have influenced the adaptation of the animal and its microbial community to the prevailing conditions over many years/centuries^15–18^.

Therefore, the purpose of this study was to determine the variability of the most common bacterial clusters and selected families in terms of physiological status (conception, early pregnancy, lambing, end of lactation) and sheep breed (Polish Lowland Sheep (PON), Świniarka Sheep (SW), synthetic line BCP).

## Material and methods

### Animals and environment

Three local native Polish breeds of sheep were included in the experiment^21,22^:

- Świniarka Sheep (SW): primitive breeds; seasonal; small-sized, poor musculature; high tolerance to environmental conditions; wool type—mixed coarse; prolificacy—120%; ewes’ weight: 25–35 kg,
- Polish Lowland Sheep (PON): seasonal; medium-sized, average meat attributes; wool type—fine; prolificacy—150%; ewes’ weight: 55–60 kg
- BCP: synthetic line sheep (37.5% PON, 12.5% fertile breed (Romanov, Finnish), 25% Berrichon du cher and 25% Charolaise); large-sized, well-defined muscles; good adaptation to the environment of eastern Poland; prolificacy—170%; ewes’ weight: 70 kg.

The study was conducted in the experimental station Bezek belonging to the University of Life Sciences in Lublin, located in the south-eastern part of Poland. The diets of ewes were formulated to their physiological status according to INRA feeding system^23^. The animals were kept in one building (each breed separated) under uniform environmental conditions (with combined airflow). Sheep are kept in the following systems: indoor (from September to mid-May), and indoor with grazing (from mid-May to early September). The mating was conducted for 6 weeks: started in September for PON, in October for SW, and in November for the BCP. Lambing state was from mid-January to February for PON, from February to March for SW and from March to April for BCP. The animals were of the same age—2 years old, and of the same sex—females. The animals did not show any disease symptoms. All animals housed in the sheepfold were fed in the same way using the feed available on the farm. In indoor period of time sheep was until May.

### Sampling

**Figure 1:**
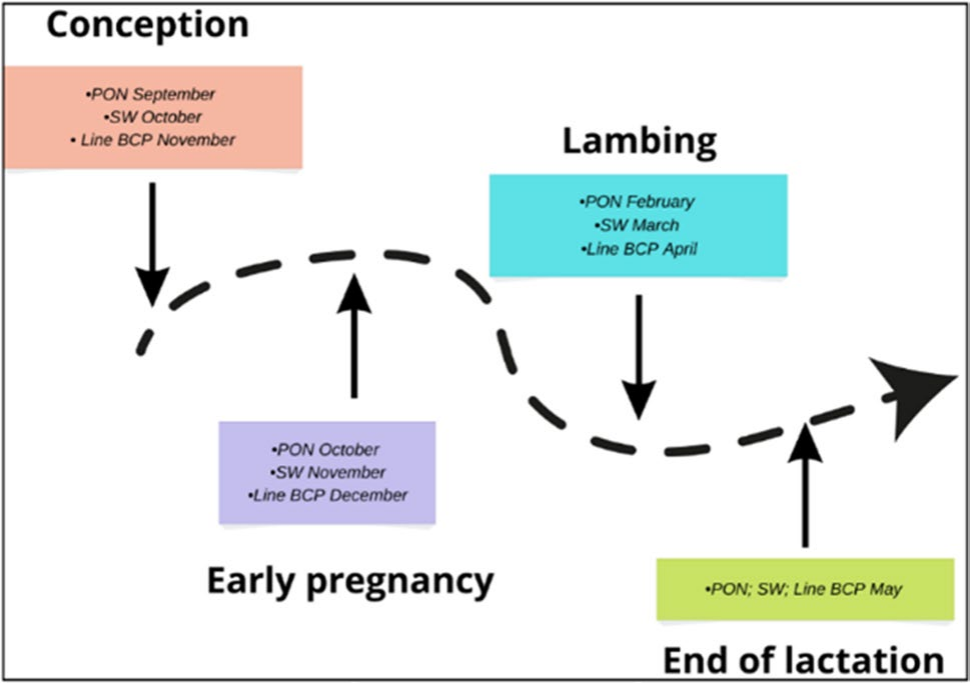
Sampling calendar. *PON* Polish Lowland Sheep, *SW* Świniarka Sheep, *Line BCP* synthetic sheep line.

From ewes of each breed (*n* = 10 animals from breed), stool samples were collected individually after excretion (up to 10 s) in 4 physiological states: conception—the mating period (C), early pregnancy (EP), lambing (L), end of lactation (EL) (Fig. 1). The samples were placed in sterile containers and then frozen at − 26 °C until analysis (30 days).

### DNA extraction

Extraction of DNA from stool was performed using the Genomic Bacteria AX Mini kit (A&A Biotechnology, Gdansk, Poland). Then, the quality of the performed DNA isolations was checked using the Thermo Scientific NanoDrop 2000 Spectrophotometer device and concentration on Qubit 4 Fluorometer (Thermo Fisher, Waltham, MA USA). The average DNA content of the samples was 40–50 μg/μL (in 50 μL). The contamination was at the level of 260/230 (contamination related to, among others, reagents used for isolation): 2.0–2.2, and 260/280 (contamination with substances such as enzymes, inhibitors): 1.8–2.0 (correct levels, according to the instruction manual of the device).

### qPCR analysis

Analysis of qPCR was performed with the use of a Bio-Rad CFX Connect 96 Touch apparatus with the SsoAdvanced™ Universal SYBR^®^ Green Supermix kit (Bio-Rad Laboratories, Inc., Irvine, CA, USA) at a volume of 10 μL in 3 technical repetitions (Table 1). A no template control (NTC-without DNA sample, only primers and water with PCR mix) test was additionally performed for each amplicon. The real-time PCR analysis strategy was based on the amplification of specific amplicons for the tested cluster (Firmicutes, Bacteroidetes, Actinobacteria and Proteobacteria) and families (*Clostridiaceae* and *Lactobacillaceae*) against the reference amplicon for all bacteria (16S rDNA) (Table 2). In addition, the obtained results were compared to the sample constituting the calibrator with the lowest level of the studied cluster and the lowest level of the reference amplicon in order to determine the relative level of DNA in terms of the tested amplicons.

**Table 1:** Proportion of PCR mix^24^.

Component	Volume per 10 μl reaction
SsoAdvanced™ Universal SYBR^®^ Green Supermix	5 μl
Forward and reverse primers	1 μl (0.8 μM)
DNA template	2 μl (0.04–0.015 × 10^–4^)
Nuclease—free water	2 μl

**Table 2:** Primers used for analysis.

Name	Primer sequence (5′ → 3′)	Forward (F)/reverse (R)	References
Universal eubacterial gene	530F (GTCCCAGCMGCNGCGG)	F	^25^
	1100R (GGGTTNCGNTCGTTG)	R	
Firmicutes	928F (TGAAACTYAAAGGAATTGACG**)**	F	^26^
	1040R (ACCATGCACCACCTGTC)	R	
Bacteroidetes	798cfbF (CRAACAGGATTAGATACCCT)	F	^26^
	cfb967R (GGTAAGGGTTCCTCGCGTAT)	R	
Actinobacteria	Eub338F (ACGGGCGGTGTGTACA)	F	^27^
	Act1159R (TCCGAGTTRACCCCGGC)	R	
Proteobacteria	27F (GAGTTTGATCMTGGCTCAG)	F	^28^
	1529R (CAKAAAGGAGGTGATCC)	R	
*Lactobacillaceae*	Lac1F (AGCAGTAGGGAATCTTCCA)	F	^29^
	Lac2SeqR (ATTTCACCGCTACACATG)	R	
*Clostridiaceae*	Clos-58-f (AAAGGAAGATTAATACCGCATAA)	F	^30^
	Clos780-r (ATCTTGCGACCGTACTCCCC)	R	

A standard curve was performed for the genes tested to determine the efficiency of each gene. A sample dilution of 10^−4^ from the 10^−2^ to 10^−7^ series of dilutions was selected for analysis. The analysis was performed according to a protocol of 40 cycles: polymerase activation and DNA denaturation 95 °C (3 min), denaturation 95 °C (15 s), annealing 60.5 °C (15 s), extension and plate reading at 72 °C (40 s). The analysis of the melting curves for the samples was performed at temperatures ranging from 65 °C (5 s) to 95 °C (0.5 °C increments in 2 s). The data were compiled using the CFX Maestro software (Bio-Rad Laboratories, Inc., Irvine, CA, USA). The efficiency of individual amplicons was correct (according to the standards established by BIO-RAD) and amounted to 89.4% for Firmicutes, 99.9% for Bacteroidetes, 91.6% for Actinobacteria, 94.2% for Proteobacteria and 98.4% for Universal primer, 94.2% for *Clostridiaceae* and 99.1% for *Lactobacillaceae*.

The data were then processed using the CFX Maestro software (Bio-Rad Laboratories, Inc., Irvine, CA, USA), where the sample with a DNA quantity of 40 μg/μL and impurity levels compliant with the above-mentioned standards was an arbitrary calibrator. Using the CFX Maestro program, the levels of the tested bacteria were calculated in relation to the amount of the reference amplicon template and the differences at the level of the studied amplicons of phyla—DNA level (ΔΔCq), taking into account the amplification efficiency of individual amplicons.

### Statistical analysis

The data was analyzed by using R statistics software (freely available) with packages “dyplr”, “ggpubr”, “FSA”, “vegan” and “devtools”. The Shapiro–Wilk test was performed—the data distribution was not normal. As a result, the analyzes were performed using PCA and the Kruskal–Wallis test (*p* < 0.05) where the factors were breed and physiology period. Results of Kruskal–Wallis test and the post hoc Dunn’s multiple comparisons test, showing significance of differences between groups. Plots were created in R using ggplot2.

### Ethics approval and consent to participate

The authors confirm that the ethical policies of the journal have been adhered to. All ewe’s were kept in accordance with the guidelines established by the Polish and European regulations regarding the welfare of farm animal^19,20^. The animals were kept in the conditions of a production farm, in accordance with the European Union Directives regarding the requirements for the use of acceptable technologies in breeding and animal welfare conditions. The studies get approval of the Local Ethical Committee for Animal Experiments based at the University of Life Sciences in Lublin (104/2015) (Act of 15 January 2015 on protection animals used for scientific or educational purposes, OJ 2015, 266, implementing the Directive 2010/63/EU of the European Parliament and the Council of 22 September 2010 on the protection of animals used for scientific purposes). Permission to use animal samples was obtained from the University of Life Sciences in Lublin—the owner of the experimental station Bezek.

## Results

### Breed influence

**Figure 2:**
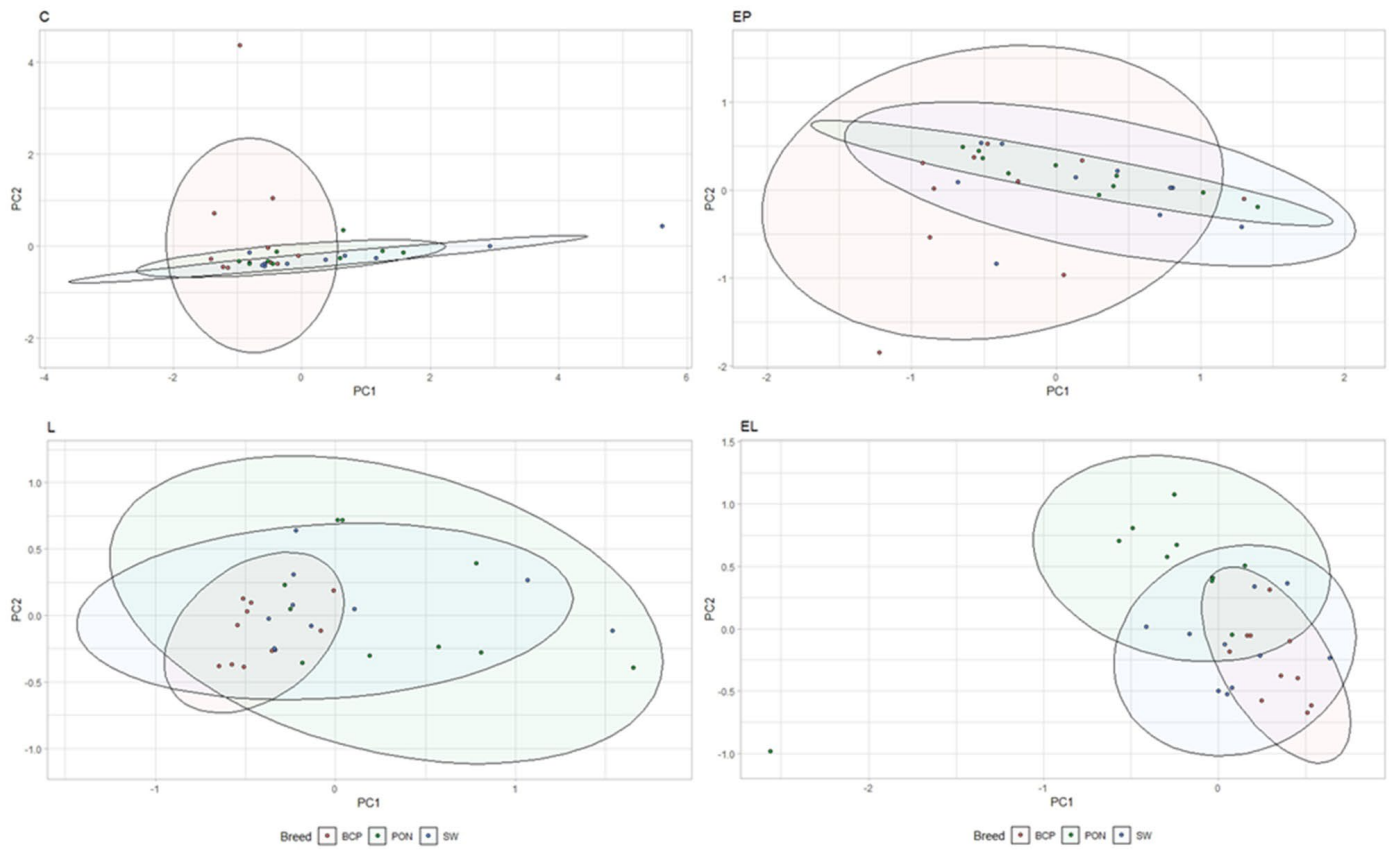
PCA results of level of bacteria depending on sheep breed (*PON* Polish Lowland Sheep, *SW* Świniarka Sheep, *BCP* synthetic sheep line BCP) in conception period.

**Figure 3:**
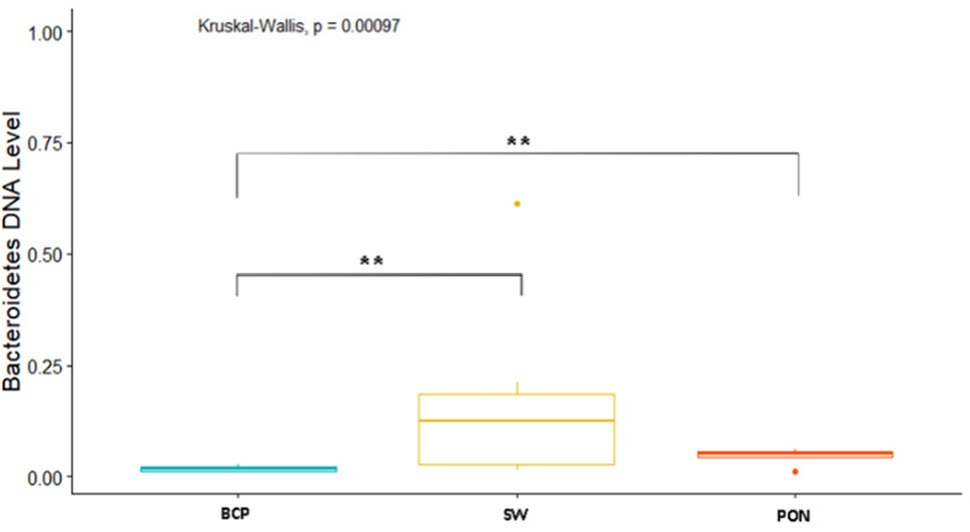
DNA level of Bacteroidetes at the conception state (C) (**p < 0.01). *PON* Polish Lowland Sheep, *SW* Świniarka Sheep, *BCP* synthetic sheep line BCP.

**Figure 4:**
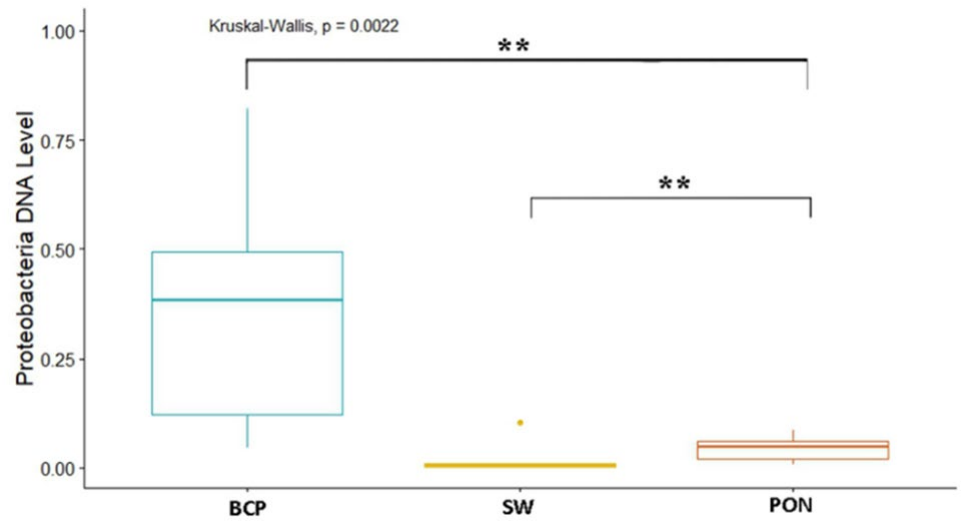
DNA level of Proteobacteria at the early pregnant state (EP) (**p < 0.01). *PON* Polish Lowland Sheep, *SW* Świniarka Sheep, *BCP* synthetic sheep line BCP.

Analysis of the levels of selected bacteria in terms of breeds in each physiological state showed variation in the levels of the studied cluster and families (*p* = 0.038) (Fig. 2). In the conception period (C), significant differences were shown in the level of the Bacteroidetes cluster (*p* < 0.05), between the BCP and SW and PON (*p* = 0.0011, *p* = 0.00073, respectively). The highest average levels were found in the samples from SW (DNA level = 0.153), while the lowest from BCP (DNA level = 0.0248) (Fig. 3). On the other hand, in the early pregnancy (EP) state, significant differences were found in the level of Proteobacteria between the BCP and SW (*p* = 0.00269) and between the PON and SW (*p* = 0.0036), where higher levels were shown in samples from the BCP line (DNA level = 0.387) (Fig. 4).

**Figure 5:**
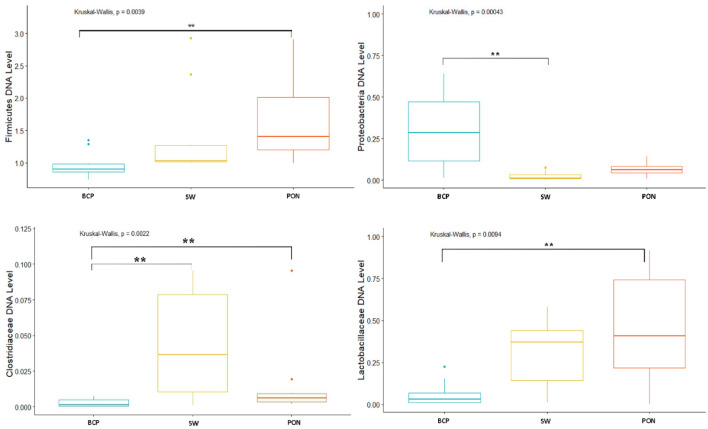
DNA level of selected analyzed bacteria at the lambing state (L) (*p < 0.05; **p < 0.01). *PON* Polish Lowland Sheep, *SW* Świniarka Sheep, *BCP* synthetic sheep line BCP.

At the lambing state (L) also showed differences in the level of the Proteobacteria cluster between the BCP and SW and (*p* = 0.00025), in addition, differences in the Firmicutes cluster were shown between samples from the BCP and PON (*p* = 0.0044) (Fig. 5). The highest level of the Proteobacteria cluster was shown in samples from the BCP (DNA level = 0.304), while the lowest from SW (DNA level = 0.0251). On the other hand, the Firmicutes cluster in samples from the PON had the highest DNA level, and from the BCP the lowest (1.24 and 0.973, respectively). There were also significant differences in the level of the *Clostridiaceae* family between the BCP and the other breeds analyzed (SW, *p* = 0.001; PON, *p* = 0.043, respectively), where the BCP had the lowest level (DNA level = 0.00276). On the other hand, the DNA level in the PON and SW was 0.0157 and 0.0427, respectively. The samples also showed differences in the level of the *Lactobacillaceae* family between the PON and BCP (*p* = 0.015) (Fig. 5).

**Figure 6:**
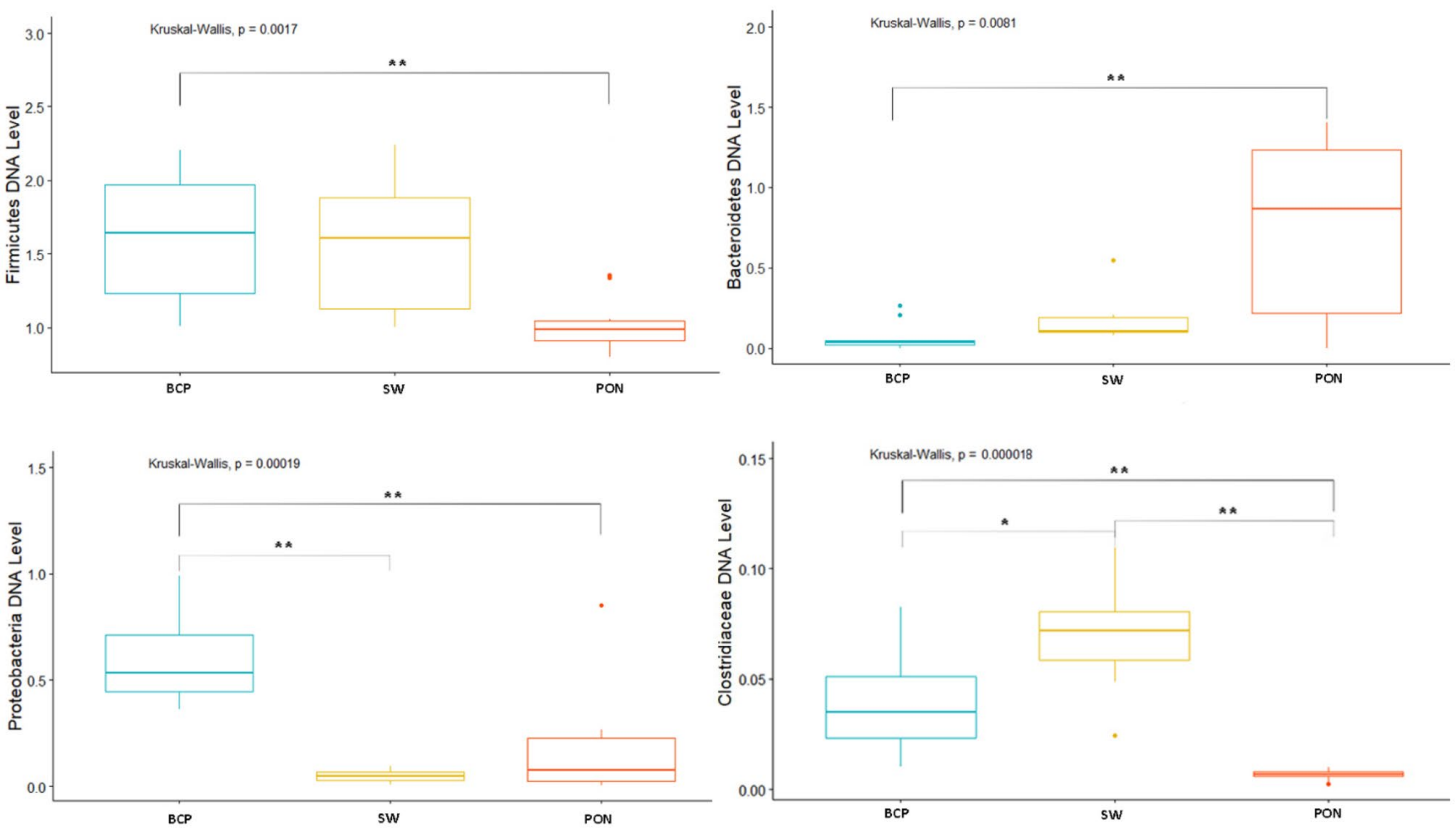
DNA level of selected analyzed bacteria at the end of lactation state (EL) (*p < 0.05; **p < 0.01). *PON* Polish Lowland Sheep, *SW* Świniarka Sheep, *BCP* synthetic sheep line BCP.

The last physiological period analyzed—end of lactation (EL) showed significant differences in the level of the Firmicutes cluster between the BCP and PON (*p* = 0.003). The DNA level of this cluster in the analyzed breeds was 1.6 for the BCP, 1.03 for the PON, and 1.55 for the SW. There were also statistical differences between the BCP and PON in the levels of the Bacteroidetes cluster (0.0675 and 0.730 DNA level, respectively), Proteobacteria (0.601 and 0.173 DNA level, respectively) and the *Clostridiaceae* family (0.383 and 0.00665 DNA level, respectively) (*p* = 0.0058, *p* = 0.0031, *p* = 0.000375, respectively). Significant differences were also found between the BCP and SW in the levels of the Proteobacteria cluster (0.601 and 0.0491 DNA level, respectively; *p* = 0.00024) and the *Clostridiaceae* family (0.383 and 0.0689 DNA level, respectively;* p* = 0.01532). In the case of the *Clostridiaceae* family, there were also significant differences between the PON and SW (*p* = 0.00001283) (Fig. 6).

### Physiological state

#### BCP line

**Figure 7:**
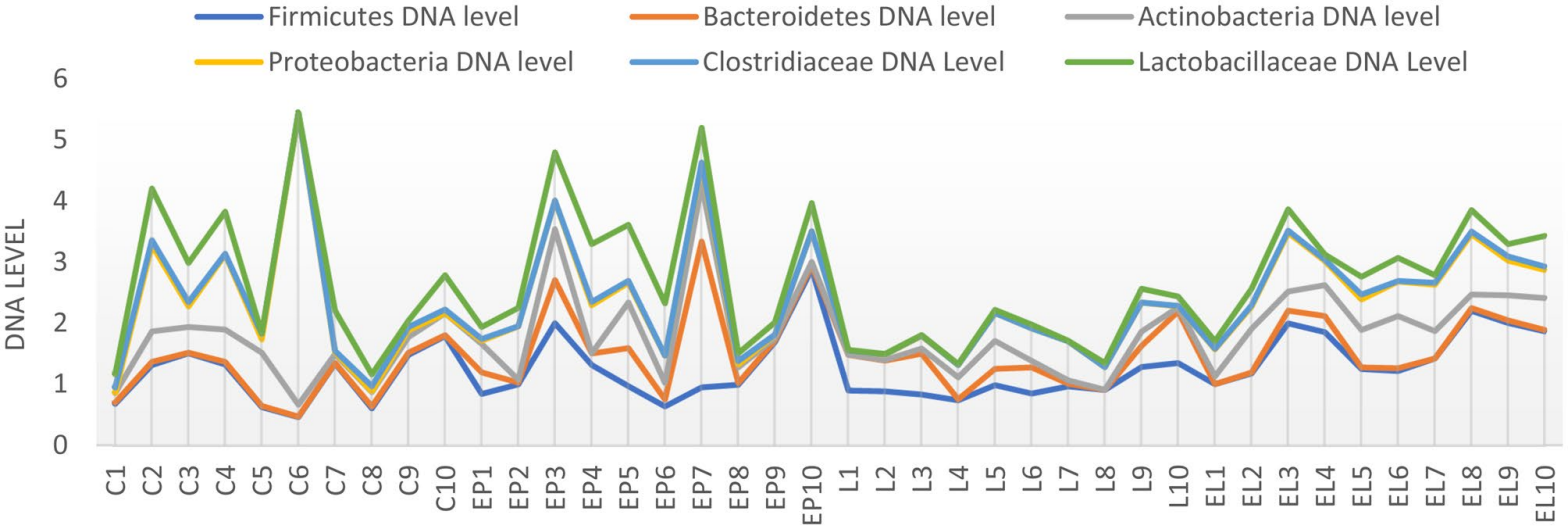
Individual DNA level of analyzed bacteria at different physiological states: *C* conception, *EP* early pregnant, *L* lambing, *EL* end of lactation (*n* = 10 animals).

**Figure 8:**
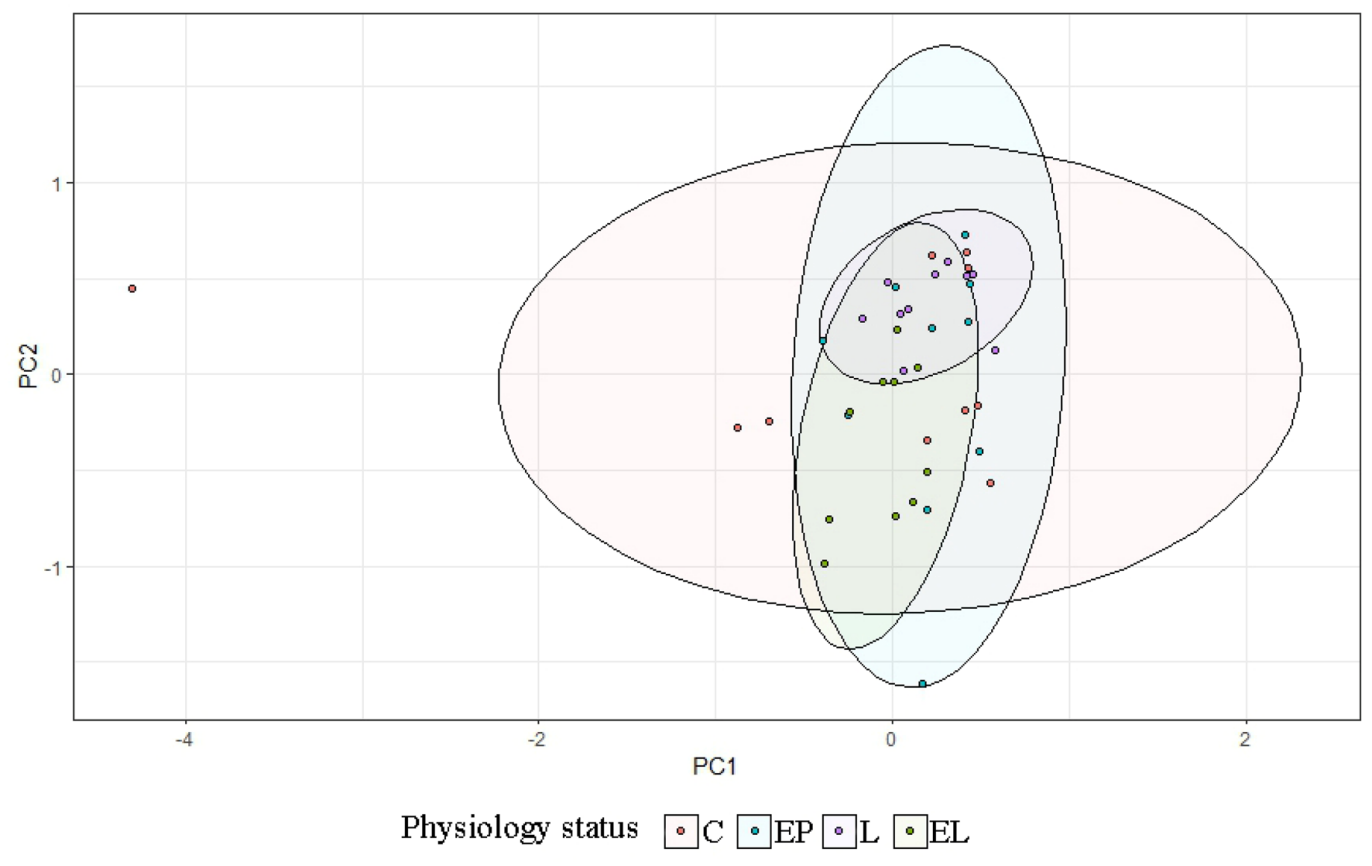
PCA results of level of bacteria depending on physiological state (*C* conception, *EP* early pregnant, *L* lambing, *EL* end of lactation).

In the case of individual analysis of the tested animals, variations are evident among individuals in the level of the tested bacteria, with the greatest variation occurring in the C and EP periods. On the other hand, in the state L the level is equalized between individuals (Fig. 7). Analysis of fecal samples from the BCP line showed a significant effect of physiological state/period on the levels of bacteria analyzed (*p* = 0.023) (Fig. 8).

**Figure 9:**
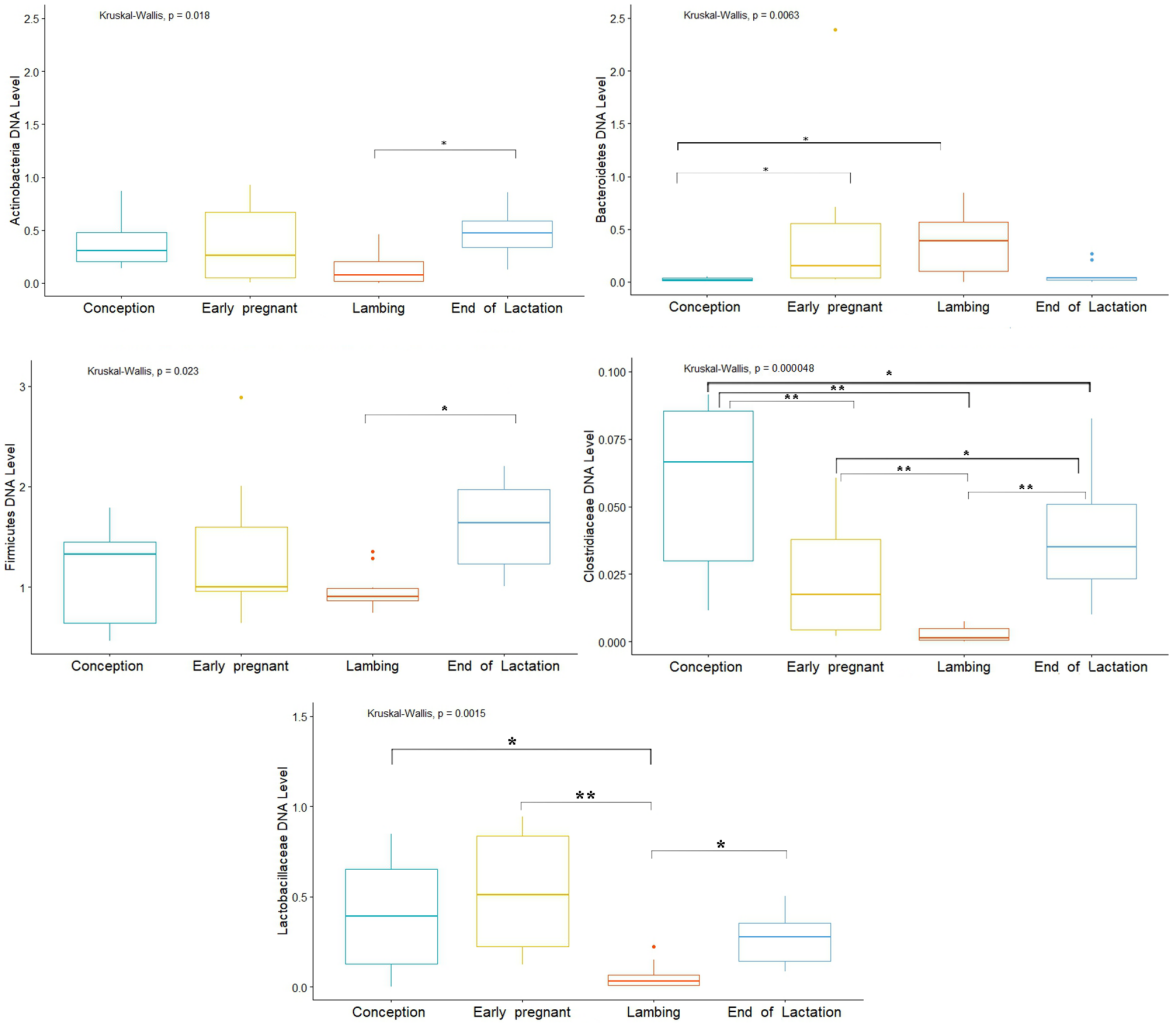
DNA level of analyzed bacteria in different physiological states (*p < 0.05; **p < 0.01).

Analysis of the Firmicutes cluster showed significant differences in levels between the EL and L periods (*p* = 0.015), where the level of the cluster in the EL state was at 1.6 and in the L state at 0.97 DNA level. The Bacteroidetes cluster, on the other hand, showed statistically significant differences between the C and EP (*p* = 0.014), and C and L periods (*p* = 0.02). DNA level of Bacteroidetes was at 0.024 in the C state, 0.36 in the EP and 0.452 in the L state. Analysis of the Actinobacteria cluster showed significant differences between the EL and L period (*p* = 0.014; DNA level 0.48 and 0.13, respectively). In contrast, there were no differences in the level of the Proteobacteria cluster according to physiological state. Analysis of selected bacterial families showed significant differences between physiological states. The *Clostridiaceae* family showed significant differences between the EL and EP, C and L periods (*p* = 0.024, *p* = 0.032, *p* = 0.00012, respectively), and also between the L and EP and C periods (*p* = 0.0037, *p* = 0.00041, respectively) and between the EP and C periods (*p* = 0.0048). The highest DNA level was shown in the C state—0.061, and the lowest level in the L state—0.002. In contrast, in the other states, the DNA level was 0.023 (EP) and 0.038 (EL). In the case of the *Lactobacillaceae* family, differences were shown between the L and C, EP and EL periods (*p* = 0.032, *p* = 0.00072, *p* = 0.0319, respectively), where the DNA levels in each state were: 0.392, 0.534, 0.061 and 0.268, respectively (Fig. 9).

#### Świniarka sheep

**Figure 10:**
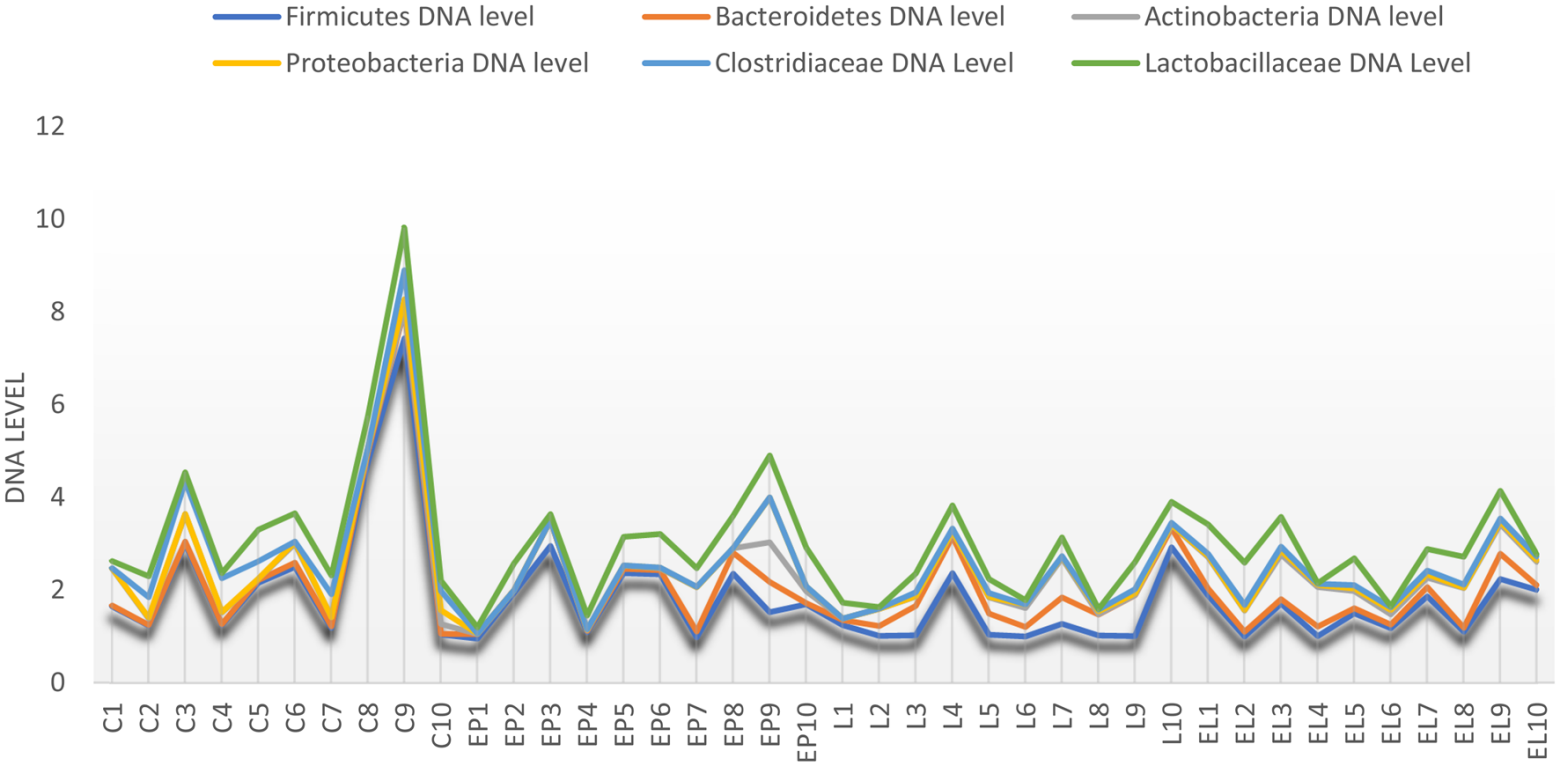
Individual DNA level of analyzed bacteria at different physiological states: *C* conception, *EP* early pregnant, *L* lambing, *EL* end of lactation (*n* = 10 animals).

**Figure 11:**
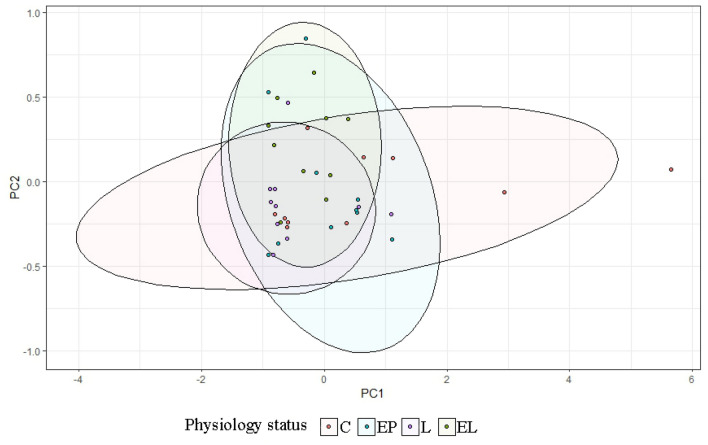
PCA results of level of bacteria depending on physiological state: *C* conception, *EP* early pregnant, *L* lambing, *EL* end of lactation.

In the case of individual analysis of the tested animals, variations are evident among individuals in the level of the tested bacteria at all periods. Analysis of fecal samples showed a significant effect of physiological state on the levels of bacteria analyzed (*p* = 0.044) (Figs. 10, 11).

**Figure 12:**
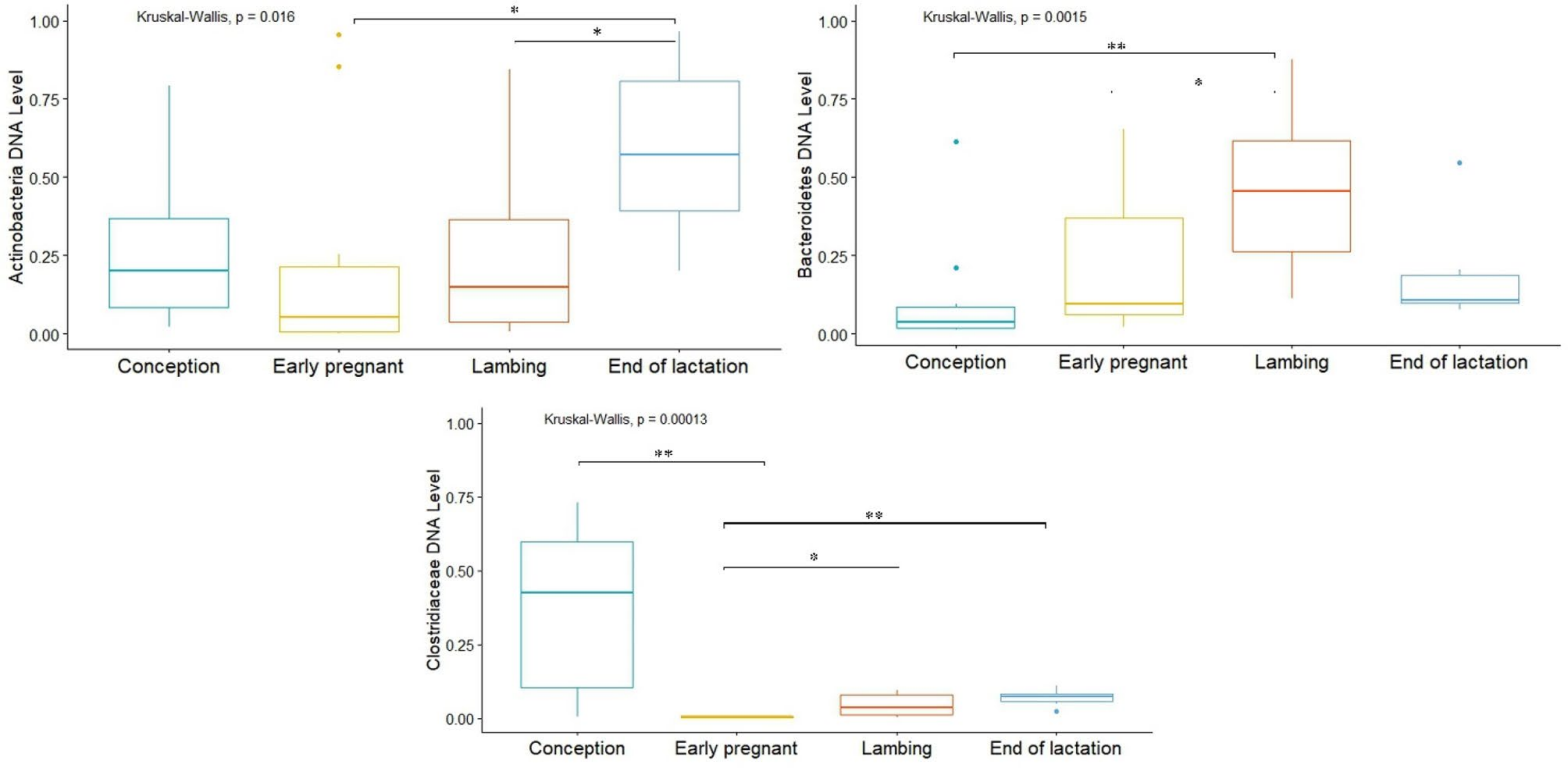
DNA level of analyzed bacteria in different physiological states (*p < 0.05; **p < 0.01).

Analysis of the bacterial clusters tested showed no significant differences in the levels of Proteobacteria and Firmicutes (range of 0.05–0.11 and 1.39–2.60 DNA level, respectively). On the other hand, the Bacteroidetes cluster showed significant differences in the levels between the C and L (*p* = 0.00061) and L and EP periods (*p* = 0.043), where the DNA level in the C state was 0.11, in the EP state was 0.17, and in the L state was 0.47. Significant differences were also shown for the Actinobacteria cluster between the EP and EL periods (*p* = 0.016), where the DNA level was 0.22 and 0.57, respectively. Additionally, were differences between EL and L (*p* = 0.041), where the DNA level was 0.23 for L period. Analysis of selected bacterial families showed significant differences for *Clostridiaceae* between the C and EP (*p* = 0.001), EL and EP (*p* = 0.00198) and EP and L (*p* = 0.048) periods. The highest DNA level was shown in state C—0.38, and the lowest in state EP—0.0044. In contrast, in the L and EL states it was 0.043 and 0.068, respectively (Fig. 12). In the case of the *Lactobacillaceae* family there were no differences in the analyzed physiological states.

#### Polish lowland sheep

**Figure 13:**
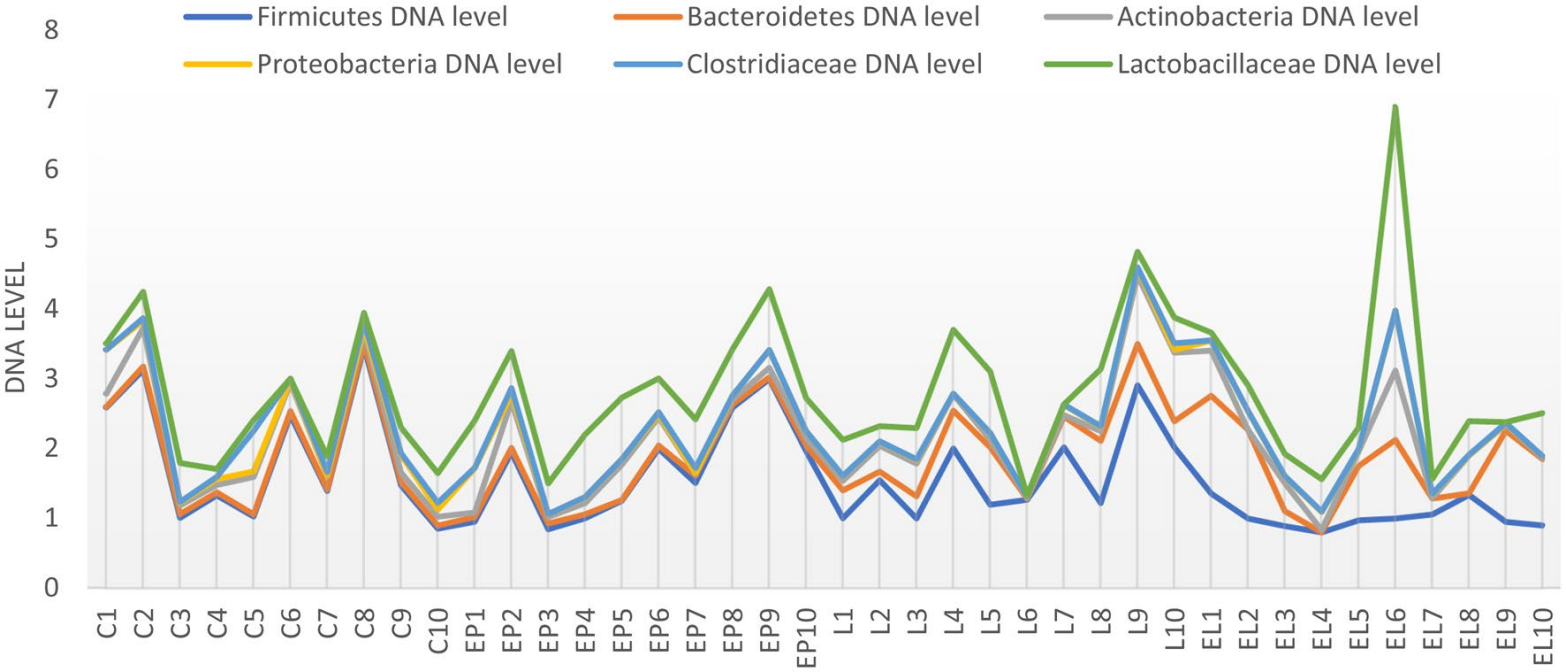
Individual DNA level of analyzed bacteria at different physiological states: *C* conception, *EP* early pregnant, *L* lambing, *EL* end of lactation (*n* = 10 animals).

**Figure 14:**
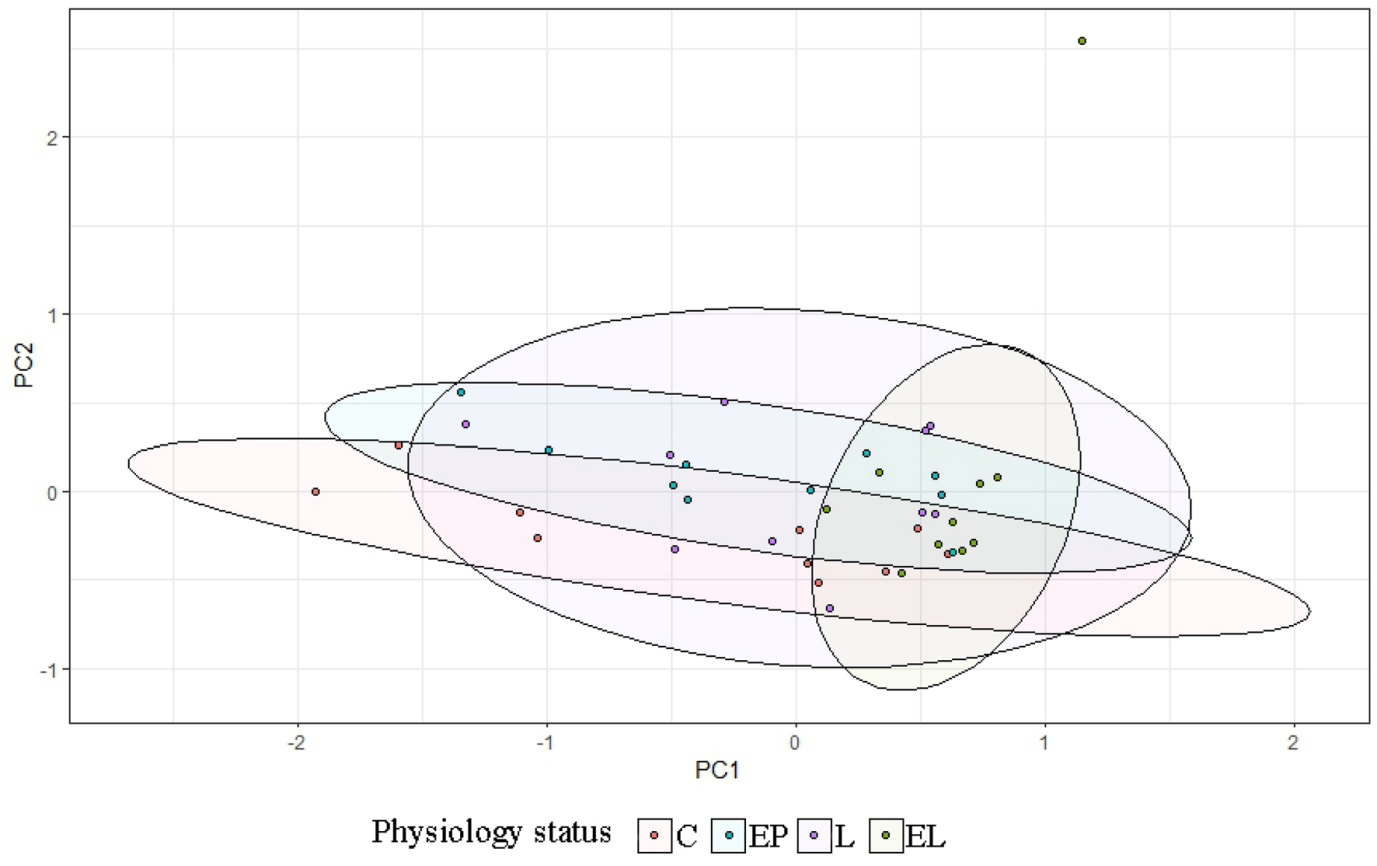
PCA results of level of bacteria depending on physiological state (*C* conception, *EP* early pregnant, *L* lambing, *EL* end of lactation).

In the case of individual analysis of the tested animals, variations are evident among individuals in the level of the tested bacteria at all periods, especially at end of lactation (EL). Analysis of fecal samples showed a significant effect of physiological state on the levels of bacteria analyzed (*p* = 0.002) (Figs. 13, 14).

**Figure 15:**
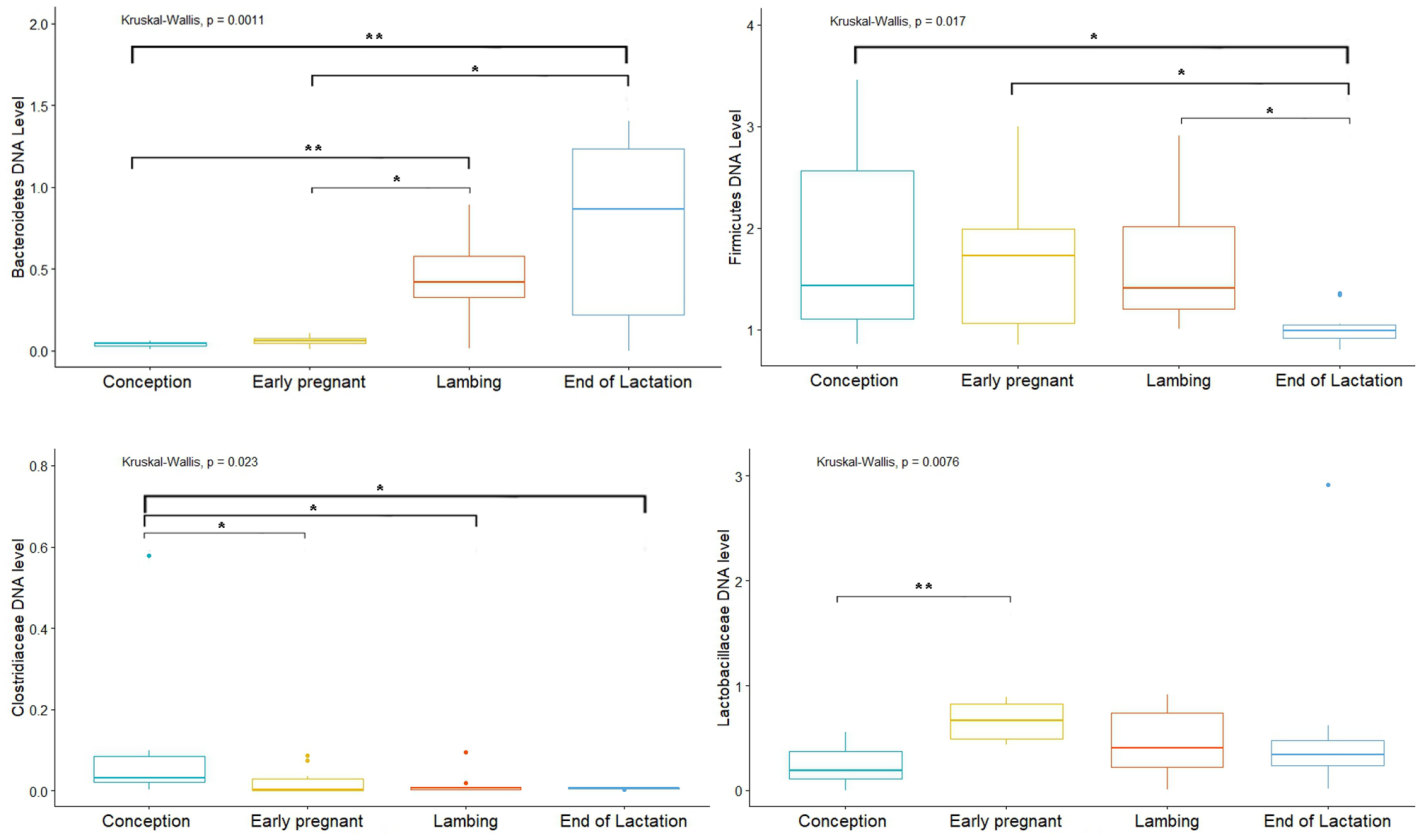
DNA level of analyzed bacteria in different physiological periods (*p < 0.05; **p < 0.01).

Analysis of Firmicutes cluster levels according to physiological state showed significant differences occurred between the EL vs EP periods (*p* = 0.036), EL vs C periods (*p* = 0.030), and EL vs L periods (*p* = 0.031), where the highest DNA level occurred during EP state—1.87, and the lowest in EL state—1.03. Significant differences were also shown in the level of the Bacteroidetes cluster between the C vs EL periods (*p* = 0.0069), C vs L (*p* = 0.0055), EP vs L (*p* = 0.0325) and EP vs EL (*p* = 0.0302), where the highest level was shown in the EL state (0.730 DNA level) and the lowest in the C state (0.0422 DNA level) (Fig. 15). In contrast, the Actinobacteria and Proteobacteria cluster showed no significant differences. Analysis of *Clostridiaceae* family levels showed significant differences between the EL and C (*p* = 0.044), EP and C (*p* = 0.0396), and C and L (*p* = 0.0382) periods, with the lowest and highest DNA level in the respectively, L and C state (0.00665 and 0.0948). In contrast, the DNA levels in the other states were 0.0219 (EP) and 0.0157 (EL). In the case of the *Lactobacillaceae* family, significant differences were shown between the EP and C periods (*p* = 0.00426), where the DNA levels in these states were 0.660 and 0.581, respectively (Fig. 15).

## Discussion

The microbial communities of the digestive system are important for ruminants in maintaining good health and production efficiency. The composition of the microbial community is not only influenced by environmental factors like diet, but also by genetic like breed^31,32^. Livestock selection is aimed at selecting breeds with important parameters for farmers, such as resistance to environmental conditions or good production parameters. There are many sheep breeds in the world, but many of which are found only regionally. In Poland there are 17 native sheep breeds including in Genetic Resources Conservation Programme^22^. The population of Polish Lowland Sheep (PON), included in the conducted study, in 2022 have consisted from 7485 animals (111 herds). Another breed included in the study was the Świniarka Sheep (SW), a primitive breed that has originally been bred in central and western Europe, representing the majority of the primitive herds at the time. As of 2022, the population in Poland was 2248 animals (34 herds)^33^. In turn, the BCP, which is a synthetic line has been bred in the 2000s in Lubelskie Voivodship^21^. The breeds included in the study differed in both type of utility (BCP—meat type, PON—meat and wool type, SW—general-purpose) and the time of origin (the oldest SW and the youngest BCP).

The study showed breed differences in the bacteria analyzed primarily between the BCP synthetic line and the other two breeds. For the BCP and PON breeds, the differences were in the level of Bacteroidetes, Proteobacteria, Firmicutes cluster and *Lactobacillaceae*, *Clostridiaceae* family. Then between BCP and SW—Bacteroidetes, Proteobacteria, Firmicutes cluster and *Clostridiaceae* family. In turn, the differences between SW and PON—Proteobacteria cluster and *Clostridiaceae* family. The noted differences may be related to the different environmental conditions of breeding and rearing these breeds over many years/centuries and different directions of use (type of utility) as indicated by the results of studies by other authors, the system of maintenance and feeding affect the adaptation of the animal and its microbial community to the prevailing conditions^15–18^. Another aspect influencing the obtained results may be the effect of the microbial community on the formation of a metabolic phenotype related to production rates, since individual bacteria and their metabolism in the digestive tract of ruminants may contribute to differences in the level of absorption of nutrients from feed^34,35^. In addition, analysis previously conducted on other three breeds of sheep have also shown significant differences in bacterial levels, suggesting a significant effect of breed on microbial community composition^24^. However, further studies on genotypic and species variations in the microbiome are recommended^12,14,36–39^.

The physiological state of the animal is an important factor affecting the microbiology of the digestive system. In the case of ewes, we can distinguish four states: conception (C), early pregnancy (EP), lambing (L), and end of lactation (EL). During the first period (C), ewes are paired with ram for copulation. The pregnancy period in sheep lasts 5 months. The young lambs are kept with ewes for a period of 30–120 days, depending on the rearing system (shortened, traditional)^8,40^. The analysis showed the occurrence of variation in the levels of the analyzed bacteria depending on the physiological period. In the case of the BCP line, there were significant individual variation, compared to the other breeds. In addition, the majority of analyzed bacteria showed significant between the L and EL periods, and between EP compared to others periods. In contrast, for the SW breed, significant changes were shown in the levels of Actinobacteria (EP and EL, L and EL), Bacteroidetes (C and L) and *Clostridiaceae* family (C and EP, EP and L, EP and EL). In contrast, the PON breed was characterized by significant variation in the levels of Firmicutes, Bacteroidetes, *Clostridiaceae* and *Lactobacillaceae,* where differences occurred mainly between the EP period and the others. The observed results between EP and other periods may be related to the body's response and preparation for fetal development^42^. In the case of females, significant changes in the microbial community were noted during pregnancy, which is probably related to changes in sex hormones^41^. Studies conducted on mice by Koren et al.^43^ showed that the microbial community in females during pregnancy changes significantly, females during this time have reduced insulin sensitivity, which prepares them to build up energy stores for the rearing of offspring. The study showed that there was a decrease in the levels of Firmicutes and Actinobacteria between the first and third trimesters of pregnancy, with a concomitant increase in the abundance of Bacteroidetes cluster. However, the performed inoculation of the microbial community of pregnant and non-pregnant mice resulted in obesity. Additionally, a study by Menon et al.^41^ indicate that sex hormone levels may play a role in gastrointestinal microbial variation. Androgens, which include testosterone, stimulate protein synthesis, gaining muscle mass at a faster rate. Estrogens, on the other hand, are associated with the stimulation of insulin-like growth factor 1 (IGF–1), estrogen during pregnancy (Estetrol—E4) additionally affects the accumulation of energy stores. In a study by Markle et al.^44^ showed that manipulating the microbiome of females by increasing testosterone levels caused a change in the qualitative and quantitative composition of the gastrointestinal tract microflora, resulting in an increase in the levels of bacteria from the Firmicutes and Bacteroidetes cluster compared to control group. The greatest increase occurred in the Firmicutes cluster (mainly increased levels of the genus *Clostridia*). In addition, due to changes in metabolic requirements, an unbalanced nutritional state can occur in later pregnancy despite constant access to feed. This may be related to the increased volume of the uterus resulting in a state of malnutrition. During such periods, both quantitative and qualitative changes may occur in the microbial community^8,45^.

## Conclusion

Conducted analyses indicate the influence of physiological state on gastrointestinal bacterial levels, including during pregnancy period. In addition, changes in bacterial levels in different physiological states indicate breed differences. The biggest differences were shown between the BCP line and the other breeds studied. Probably it was related to the period of its development and the time of selection to obtain the expected properties (BCP—2000s, Polish Lowland Sheep—1963, Świniarka Sheep—before 1921). However, both of these factors requires further and more detailed analysis.

## Data Availability

The datasets used and/or analysed during the current study available from the corresponding author on reasonable request. All data generated or analysed during this study are included in this published article.
